# Diabetes mellitus and 10-year clinical outcomes after percutaneous coronary intervention with drug-eluting stents: insights from the DECADE cooperation

**DOI:** 10.1186/s12933-026-03434-3

**Published:** 2026-11-11

**Authors:** Maddalena Immobile Molaro, Tobias Lenz, Lorenz Räber, J. J. Coughlan, Salvatore Brugaletta, Fiorenzo Simonetti, Luis Ortega-Paz, Karl-Ludwig Laugwitz, Lisette Okkels Jensen, Dik Heg, Kevin Kris Warnakula Olesen, Stephan Windecker, Manel Sabaté, Michael Maeng, Adnan Kastrati, Salvatore Cassese

**Affiliations:** 1 Klinik für Herz- und Kreislauferkrankungen, TUM Klinikum Deutsches Herzzentrum, Technische Universität München, Lazarettstrasse 36, 80636 Munich, Germany; 2 Department of Advanced Biomedical Sciences, Federico II University of Naples, Naples, Italy; 3 Department of Cardiology, Inselspital, Bern University Hospital, University of Bern, Bern, Switzerland; 4 Department of Cardiology and Cardiovascular Research Institute, Mater Private Network, Dublin, Ireland; 5 School of Pharmacy and Biomolecular Sciences, RCSI University of Medicine and Health Sciences, Dublin, Ireland; 6 Department of Cardiology, Institut d’Investigacions Biomèdiques August Pi i Sunyer, Hospital Clínic, University of Barcelona, Barcelona, Spain; 7 CIBER-CV: Biomedical Research Networking Center in Cardiovascular Diseases, Madrid, Spain; 8 Division of Cardiology, University of Florida College of Medicine, Jacksonville, FL USA; 9 Medizinische Klinik und Poliklinik, Klinikum rechts der Isar, Technische Universität München, Munich, Germany; 10 Deutsches Zentrum für Herz- und Kreislauf-Forschung (DZHK) e.V. (German Centre for Cardiovascular Research), Partner Site Munich Heart Alliance, Munich, Germany; 11 Department of Cardiology, Odense University Hospital, Odense, Denmark; 12 Department of Clinical Research, University of Bern, Bern, Switzerland; 13 Department of Cardiology, Aarhus University Hospital, Aarhus, Denmark; 14 Department of Cardiology, Hospital Universitari de Bellvitge, BIO-HEART Cardiovascular Diseases Research Group, IDIBELL (Bellvitge Biomedical Research Institute) L’Hospitalet de Llobregat, Barcelona, Spain; 15 University of Barcelona, Barcelona, Spain

**Keywords:** Coronary artery disease, Diabetes mellitus, Drug-eluting stent, Long-term outcomes, Percutaneous coronary intervention

## Abstract

**Background:**

Diabetes mellitus (DM) is a well-established risk factor for adverse cardiovascular outcomes after percutaneous coronary intervention (PCI). However, research assessing the magnitude and the timing of clinical events over the long term in patients with DM undergoing PCI with a drug-eluting stent (DES) is limited.

**Methods:**

The data of patients treated with DES in five randomized controlled trials with available 10-year follow-up were pooled. Patients were grouped according their DM diagnosis at baseline. The primary endpoint was all-cause death. The secondary endpoints were cardiovascular death, myocardial infarction (MI), definite stent thrombosis (ST), target lesion revascularization (TLR), target vessel revascularization (TVR) and non-target vessel revascularization (nTVR). All-cause death was estimated using the Kaplan–Meier method, whereas cumulative incidence functions accounting for the competing risk of death were calculated for secondary endpoints. Associations were evaluated using both cause-specific Cox regression, yielding cause-specific hazard ratios (HRs), and Fine-Gray regression, yielding subdistribution hazard ratios (sHRs). Multivariable analyses with adjustment for relevant variables and subgroup analyses were performed.

**Results:**

Among 9,699 patients, 2,298 had DM at baseline. Compared with patients without DM, patients with DM had a higher risk of all-cause death (adjusted hazard ratio [HR_adj_] 1.68 [95% confidence interval 1.59–1.78]) and cardiovascular death (HR_adj_ 1.74 [1.63–1.87]) up to 10 years. The risks of definite ST (HR_adj_ 1.55 [1.18–2.04]), TLR (HR_adj_ 1.35 [1.21–1.51]), TVR (HR_adj_ 1.28 [1.14–1.43]), and nTVR (HR_adj_ 1.23 [1.09–1.40]) were significantly increased in patients with DM. Landmark analyses showed endpoint-specific temporal patterns: the excess MI risk was predominantly observed within the first month, whereas the excess definite ST risk was detected between 30 days and 1 year. Notably, the association between DM at baseline and clinical outcomes varied across subgroups defined by age, sex, clinical presentation, and DES-generation (all P_interaction_ <0.001). Insulin-treated patients represent the higher risk phenotype.

**Conclusions:**

DM was associated with distinct temporal patterns of adverse outcomes after PCI with DES, with an early associations with MI and definite ST, as well as an increased risk of death and repeat revascularisation in the longer term. These findings further emphasize the need for intensified secondary prevention and provide a long-term benchmark against which novel management strategies should be evaluated in patients with DM undergoing PCI.

**Graphical Abstract:**

**Figure:**
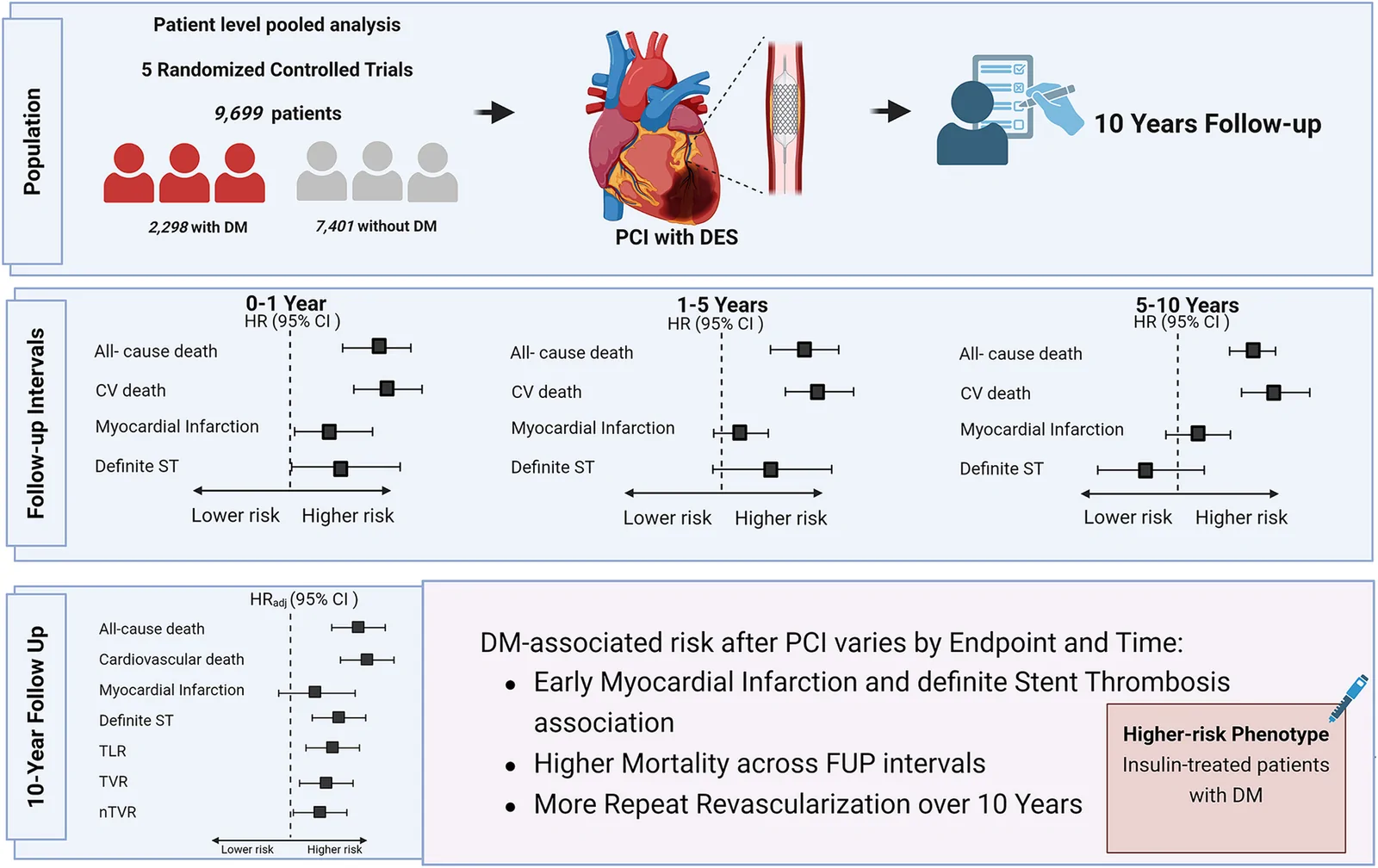

**Supplementary Information:**

The online version contains supplementary material available at https://doi.org/10.1186/s12933-026-03434-3.

## Research insights

**What is currently known about this topic?**

Diabetes mellitus is associated with adverse outcomes after percutaneous coronary intervention (PCI).

**What is the key research question?**

What is the impact of diabetes on mortality, ischemic events, stent-related outcomes, and coronary disease progression over 10 years after PCI with DES?

**What is new?**

In this individual patient-data analysis of 9,699 participants from five randomized trials, diabetes was associated with persistently increased risks of all-cause and cardiovascular death, definite stent thrombosis, and repeat revascularization involving both target and non-target vessels. The excess risk of myocardial infarction was observed in the first month after PCI. Its association with outcomes varied according to age, sex, clinical presentation, DES generation and insulin treatment.

**How might this study influence clinical practice?**

PCI addresses focal coronary lesions but does not eliminate the long-term systemic cardiovascular risk associated with diabetes. Findings support prolonged surveillance and intensive, individualized secondary prevention after PCI, including comprehensive risk-factor management and contemporary glucose-lowering therapies with proven cardiovascular benefits. The findings characterize endpoint-specific temporal patterns of risk after PCI in patients with DM and highlight a long-term burden extending beyond the treated lesion. The results also provide a long-term benchmark for evaluating whether newer diabetes treatments improve outcomes after PCI.

**Supplementary Information:**

The online version contains supplementary material available at https://doi.org/10.1186/s12933-026-03434-3.

## Introduction

Diabetes mellitus (DM) is a well-known risk factor for both the development of coronary artery disease (CAD) and poor clinical outcomes even after percutaneous coronary intervention (PCI) with drug-eluting stent (DES) implantation [1–4]. Altered glucose and lipid control contribute to an increased thrombogenic pathway and the progression of coronary atherosclerosis, providing potential mechanistic explanations [5].

Several trials investigating the performance of different DES platforms have conducted in-depth analyses of clinical outcomes in patients with and without DM, given the high-risk features of patients with DM [6]. In addition, although some previous investigations have been specifically designed to evaluate the performance of DES in patients with DM [7, 8], there is a paucity of data concerning 10-year outcomes of patients undergoing PCI with DES [9]. In fact, the majority of randomized controlled trials have follow-up durations of approximately five years, which may be insufficient to fully evaluate the effectiveness of a percutaneous treatment strategy, particularly for patients with DM [10, 11].

Therefore, the present analysis reports the 10-year clinical outcomes of patients enrolled in five large-scale randomized trials of DES, stratified according to the diagnosis of DM at the time of the index PCI.

## Methods

The data collection methods and study description have been published previously [12]. The DECADE cooperation (Adverse Events and Coronary Artery Disease Progression) is an individual patient-level pooled analysis of DES trials each with 10-year follow-up. The included trials were EXAMINATION (Clinical Evaluation of the Xience-V Stent in Acute Myocardial Infarction), ISAR-TEST 4 (Intracoronary Stenting and Angiographic Results: Test Efficacy of 3 Limus-Eluting Stents), ISAR-TEST 5 (Intracoronary Stenting and Angiographic Results: Test Efficacy of Sirolimus- and Probucol-Eluting Versus Zotarolimus-Eluting Stents), SIRTAX (Sirolimus-Eluting Versus Paclitaxel-Eluting Stents for Coronary Revascularization) and SORT OUT III (Randomized Clinical Comparison of the Endeavor and the Cypher Coronary Stents in Non-Selected Angina Pectoris Patients). Details on the design, characteristics of enrolled patients, and 10-year outcomes of these trials have been published previously [13–17]. In all the included trials, post-PCI antithrombotic therapy following DES implantation consisted of lifelong aspirin combined with clopidogrel for 6 to 12 months for those patients not requiring lifelong anticoagulation. Each study included in the present analysis was approved by the institutional review board or ethics committee at each participating center, and all patients provided signed, written informed consent before receiving the assigned treatment.

### Outcomes

The primary endpoint of this analysis was all-cause death. The secondary endpoints included cardiovascular death, myocardial infarction (MI), definite stent thrombosis (ST), target lesion revascularization (TLR), target vessel revascularization (TVR), and non-target vessel revascularization (nTVR), which were assessed over a follow-up period of up to 10 years. All outcomes were defined according to the definitions reported in the original protocols of included trials (Supplemental Table 1). The outcomes data were collected and entered into a common data sheet in the form in which they were reported in the original trials. For the current analysis, patients were dichotomized according to whether they had or not a diagnosis of DM at baseline. This was determined via information recorded in the electronic case report forms at the time of randomization in all included trials. Patients were categorized as insulin-treated if the electronic case report forms indicated baseline use of insulin (either alone or in combination with other oral antidiabetic medications). Outcome data were available for up to 10 years of follow-up across all trials except in the SORT OUT III, for which data on definite ST were only available for up to 5 years [15].

### Statistical analysis

Continuous variables are reported as median and interquartile range (IQR) and were compared using the Wilcoxon rank-sum test. Categorical variables are presented as counts and percentages and were compared using the χ² test or Fisher’s exact test, as appropriate.

For outcomes subject to competing risks, cumulative incidences were estimated nonparametrically using the Aalen-Johansen estimator. Associations were evaluated using both cause-specific Cox regression, yielding cause-specific hazard ratios (HRs), and Fine-Gray regression, yielding subdistribution hazard ratios (sHRs). For nonfatal outcomes, death was considered a competing event, whereas for cause-specific mortality outcomes, death from other causes was considered the competing event. For all-cause death, event probabilities were estimated using the Kaplan-Meier method and associations using Cox proportional hazards regression. Multivariable analyses were performed using both cause-specific Cox proportional hazards and Fine-Gray subdistribution hazard regression models, with the same set of prespecified covariates included in both models. Patients with missing values for any of the covariates included in the model were not retained in the multivariable analyses. Adjusted cause-specific hazard ratios (HR_adj_s) and adjusted subdistribution hazard ratios (sHRs), respectively, with corresponding 95% confidence intervals (CIs), were reported. The cause-specific models estimated associations with the instantaneous rate of the event among individuals remaining at risk, whereas the Fine-Gray models estimated associations with the cumulative incidence of the event in the presence of competing events. Landmark analyses were performed across three follow-up intervals (0–1, 1–5, and 5–10 years after PCI) to evaluate the association between DM and mortality outcomes and to characterize differences in risk at early, intermediate and late stages. Because the proportional hazards assumption was not met in the analyses of some outcomes of interest, we performed landmark analyses for these outcomes, with a landmark at 30-day and/or 1-year follow-up.

Unadjusted trial-specific hazard ratios (HRs) and their 95% confidence intervals were estimated for each endpoint using Cox proportional hazards models and Fine-Gray regression models. Moreover, to assess between-trial heterogeneity in the association between DM and all-cause death, trial-specific hazard ratios (HRs) were pooled across the five trials using inverse-variance meta-analysis on the log-HR scale. Pooled HRs and corresponding 95% confidence intervals were calculated using both common (fixed)-effect and random-effects models. For the random-effects model, between-trial variance (τ²) was estimated using restricted maximum likelihood, with its 95% confidence interval calculated using the Q-profile method. Heterogeneity was assessed using Cochran’s Q test and quantified using the I² statistic.

The outcomes of interest were explored in subgroups according to age, sex, clinical presentation (acute coronary syndrome, ACS vs. chronic coronary syndrome), and DES generation, with interaction terms tested to assess the heterogeneity in the association between baseline DM status and clinical outcomes. In addition, all outcomes of interest were assessed in patients with DM according to whether or not they received insuli​n therapy at admission. All the statistical tests were two-sided, and a P value < 0.05 was considered statistically significant. Statistical analysis was performed with the R Statistical Package (R Foundation for Statistical Computing, Vienna, Austria).

## Results

### Baseline and procedural characteristics

We analysed 9,699 patients, including 2,298 with DM and 7,401 without DM. Table 1 reports the baseline clinical characteristics, and Supplemental Table 2 displays the distribution of patients according to baseline diagnosis of DM across the trials included in the DECADE cooperation, along with the types of DES platforms evaluated. The median follow-up was 10.0 years in both groups. Trial-specific median follow-up duration and the number and proportion of patients lost to follow-up at 1, 5, and 10 years, according to diabetes status, are reported in Supplemental Table 3. The proportion of men, the median age and the body mass index were higher among patients with DM than among those without DM. The DM cohort presented a higher prevalence of standard modifiable cardiovascular risk factors, including hypertension and hypercholesterolemia, whereas active smoking was less common. A history of prior MI was more frequent in patients with DM. Patients with DM exhibited advanced CAD, with a higher prevalence of three-vessel involvement. Although the number of treated lesions was comparable between groups, patients with DM more frequently presented with complex coronary anatomy, characterized by a greater proportion of type B2/C lesions according to the ACC/AHA classification [18]. In addition, the DM cohort had smaller reference vessel diameters, required a longer total stent length​, and presented higher residual diameter stenosis after index PCI (Supplemental Table 4).

**Table 1:** Clinical characteristics as per DM diagnosis at baseline

Characteristics	Patients with DM (*n* = 2,298)	Patients without DM (*n* = 7,401)	*P* value
Age, y	68.0 (60.5–74.4)	65.9 (57.0–73.5)	< 0.001
Sex, n (%)			< 0.001
* Female*	608 (26.5)	1,688 (22.8)	
Body mass index, kg/m^2^	28.3 (25.5–31.4)	26.6 (24.3–29.3)	< 0.001
Smoker, n (%)	438 (19.3)	1927 (26.6)	< 0.001
Hypertension, n (%)	1,697 (74.1)	4,226 (57.7)	< 0.001
Oral antidiabetic therapy, n (%)	825 (50.8)	0 (0.0)	< 0.001
Insulin therapy, n (%)	635 (27.6)	0 (0.0)	< 0.001
Hypercholesterolemia, n (%)	1,539 (67.2)	4,571 (62.4)	< 0.001
Ejection fraction, %	55.0 (43.0–61.0)	56.0 (48.0–62.0)	< 0.001
Previous MI (%)	659 (28.8)	1,888 (25.8)	0.005
Clinical presentation, n (%)			< 0.001
* Stable Angina*	1,281 (55.7)	3,862 (52.2)	
* Unstable Angina*	397 (17.3)	921 (12.4)	
* Acute MI*	620 (27.0)	2,618 (35.4)	
* Total ACS*,* n (%)*	1,017 (44.3)	3,539 (47.8)	0.003
Number of lesions	1.0 (1.0–2.0)	1.0 (1.0–2.0)	0.295
CAD, n (%)			< 0.001
* 1-vessel disease*	423 (21.6)	1,834 (33.9)	
* 2-vessel disease*	441 (22.5)	1,357 (25.1)	
* 3-vessel disease*	1,091 (55.6)	2,206 (40.8)	

Data are shown as counts (%) or median (interquartile range). Data were analyzed at a patient-level. ACS= acute coronary syndrome; CAD= coronary artery diasease; MI= myocardial infarction

### Clinical outcomes

**Fig. 1:**
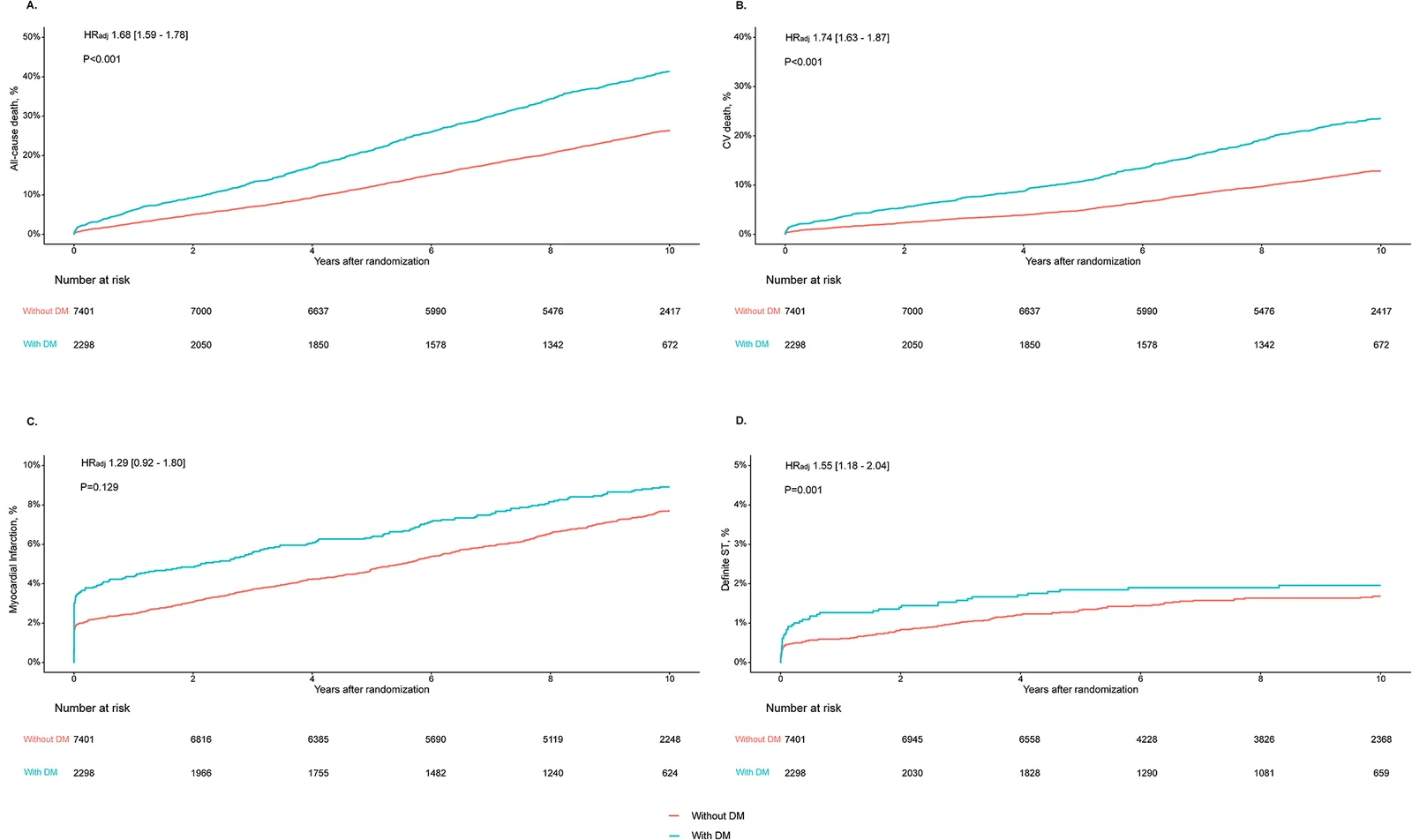
Ten-year cumulative incidence of all-cause death, cardiovascular death, MI, definite ST according to DM diagnosis at baseline. Cumulative incidence function curves and subdistribution adjusted hazard ratio (HR_adj_) with accompanying 95% confidence interval (CI) for all-cause death (**Panel A**), CV death (**Panel B**), MI (**Panel C**) and definite ST (**Panel D**) according DM diagnosis at baseline. Adjusted curves are derived from Cox/Fine and Gray model. CV= cardiovascular; DM= diabetes mellitus; MI= myocardial infarction; ST= stent thrombosis

Clinical outcomes up to the 10-year follow-up are reported in Table 2 and Supplemental Tables 5–6 for both the unadjusted and adjusted analyses. At 10-year follow-up after PCI with DES, all-cause death, was observed in 894 of 2,298 (41.3%) patients with DM and in 1,875 of 7,401 (26.4) patients without DM. After multivariable adjustment, DM was associated with a significantly higher risk of all-cause death through the 10-year follow-up (HR_adj_ 1.68 [1.59–1.78]; Fig. 1A).

**Table 2:** Ten-year clinical outcomes as per DM diagnosis at baseline

Endpoint	Patients with DM (*n* = 2,298)	Patients without DM (*n* = 7,401)	HR [95% CI]	*P* value	HR_adj_ [95% CI]	*P* value
All-cause death, n(%)	894 (41.3)	1,875 (26.4)	1.77 [1.63–1.91]	< 0.001	1.68 [1.59–1.78]	< 0.001
Cardiovascular death, n(%)	507 (23.5)	912(12.9)	2.07 [1.85–2.30]	< 0.001	1.74 [1.63–1.87]	< 0.001
Noncardiovascular death, n(%)	387 (17.8)	963 (13.5)	1.48 [1.31–1.66]	< 0.001	1.58[1.36–1.84]	< 0.001
Myocardial infarction, n(%)	198 (8.9)	554 (7.7)	1.28 [1.08–1.50]	0.003	1.29 [0.92–1.80]	0.129
Definite ST, n(%)	44 (2.0)	116 (1.7)	1.28 [0.90–1.80]	0.167	1.55 [1.18–2.04]	0.001
Target lesion revascularization n(%)	417 (18.6)	984 (13.5)	1.52 [1.35–1.70]	< 0.001	1.35 [1.21–1.51]	< 0.001
Target vessel revascularization, n(%)	530 (23.5)	1,295 (17.7)	1.46 [1.32–1.61]	< 0.001	1.28 [1.14–1.43]	< 0.001
Non-target vessel revascularization, n(%)	556 (24.8)	1,244 (17.0)	1.63 [1.47–1.80]	0.003	1.23 [1.09–1.40]	0.001

Unadjusted and adjusted hazard ratios (HRs) with corresponding 95% confidence intervals (CIs), were derived from standard Cox proportional hazards models for all-cause death and cause-specific Cox models for the other endpoints. Multivariable models were adjusted for age, body mass index, sex, smoke, DES generation, hypertension, hypercholesterolemia, prior myocardial infarction, acute coronary syndrome presentation, multivessel disease, and treatment of the left anterior descending coronary artery, with robust standard errors with clustering for trial. HR: hazard ratio; HRadj: adjusted hazard ratio; ST: stent thrombosis; CI: confidence interval. Values are n (%)

Cardiovascular death occurred in 507 of 2,298 (23.5) patients with DM and in 912 of 7,401 (12.9) patients without DM. After multivariable adjustment, DM was associated with a significantly higher risk of cardiovascular death (HR_adj_: 1.74 [1.63–1.87]; Fig. 1B).

MI occurred in 198 of 2,298 (8.9) patients with DM and in 554 of 7,401 (7.7) patients without DM. After multivariable adjustment, DM was associated with a numerically higher, although not significantly increased​ risk of MI (HR_adj_ 1.29 [0.92–1.80]; Fig. 1C).

Definite ST was an infrequent event, occurring in 44 of 2,298 (2.0) patients with DM and 116 of 7,401 (1.7) patients without DM. Following adjustment, DM was found to be associated with an increased risk of definite ST (HR_adj_ 1.55 [1.18–2.04]; Fig. 1D).

Overall, repeat revascularization events occurred significantly more often in patients with DM as compared to patients without. In fact, TLR occurred in 417 of 2,298 (18.6) patients with DM and 984 of 7,401 (13.5) patients without DM (HR_adj_1.35 [1.21–1.51]; Supplemental Fig. 1A). Consistently, patients with DM were at higher risk of TVR (HR_adj_1.28 [1.14–1.43]) and nTVR (HR_adj_1.23 [1.09–1.40]) (Supplemental Fig. 1B and Supplemental Fig. 1C) as compared to patients without DM.

### Landmark analysis

The Supplemental Table 7 presents the results of the Schoenfelds global goodness-of-fit test for the outcomes of interest. The landmark analyses aimed to explore the temporal pattern of risk over a 10-year period for outcomes that did not meet the proportional hazards assumption. Patients with DM displayed an almost two-fold increased risk of all-cause death from 0 to 1 year after the index procedure (HR 2.21 [1.85–2.64]), with a lower though still significantly increased risk between 1 and 5 years (HR 1.79 [1.48–2.06]) and between 5 and 10 years (HR 1.67 [1.49–1.86]; all *P* < 0.001; Supplemental Fig. 2A).

A similar temporal pattern and magnitude of risk were observed for cardiovascular death with a markedly increased risk from 0 to 1 year after the index procedure in patients with DM as compared to those without (HR 2.41 [1.91–3.05]). The landmark analysis of patients who were still alive after 1 year revealed that DM was associated with a significantly higher risk of cardiovascular death between 1 and 5 years (HR 2.25 [1.74–2.91]), and between 5 and 10 years (HR 1.90 [1.46–2.46]; all *P* < 0.001; Supplemental Fig. 2B).

Patients with DM displayed a higher risk of MI between 0 and 1 year after the index procedure (HR 1.77 [1.11–2.84]), an effect largely driven by events occurring within the first 30 days after index PCI (HR 1.80 [1.13–2.88]; Supplemental Fig. 3). In contrast, the risk of MI did not increase significantly between 1 and 5 years (HR 0.97 [0.85–1.10]) or between 5 and 10 years (HR 1.03 [0.89–1.19]; Supplemental Fig. 2C). At 1 year, diabetes was associated with an approximately two-fold higher risk of definite ST, although the association did not reach conventional statistical significance (HR 2.10 [0.99–4.43]; Supplemental Fig. 2D). Landmark analyses demonstrated that this excess risk was primarily driven by events occurring between 30 days and 1 year after PCI, during which patients with DM experienced a more than three-fold higher risk of definite ST (HR 3.30 [1.65–6.57]; Supplemental Fig. 4). No statistically significant association was detected between 1 and 5 years (HR 0.85 [0.52–1.41]) or between 5 and 10 years (HR 0.37 [0.12–1.18]; Supplemental Fig. 2D).

### Trial-specific estimates and between-trial heterogeneity for the primary outcome

Trial-specific clinical outcomes and hazard ratios according to diabetes status are presented in Supplemental Tables 8–12. In a two-stage meta-analysis of trial-specific estimates for all-cause death, DM was associated with higher mortality in each of the five trials, with HRs ranging from 1.57 to 2.33. The pooled random-effects HR was 1.70 (1.57–1.85; *P* < 0.001), with a similar estimate from the common-effect model. No statistically significant between-trial heterogeneity was detected (Cochran’s Q = 6.16; *P* = 0.188), although the uncertainty around heterogeneity was moderate (I²=35.0%; Supplemental Fig. 5).

### Ten-year clinical outcomes in patients with DM according to insulin-therapy

Among patients with DM, nearly one fourth (*n* = 635) received insulin-therapy. Supplemental Table 13 reports the baseline clinical characteristics of patients with DM as per insulin treatment. Compared with noninsulin-treated patients, insulin-treated patients had a significantly higher risk of all-cause death, cardiovascular death, TLR, and TVR, whereas the risks of MI, definite ST, and nTVR were not significantly different. The complete list of risk estimates for these outcomes according to insulin-therapy is displayed in Supplemental Table 14.

### Ten-year clinical outcomes according to age, sex, clinical presentation and DES type

**Fig. 2:**
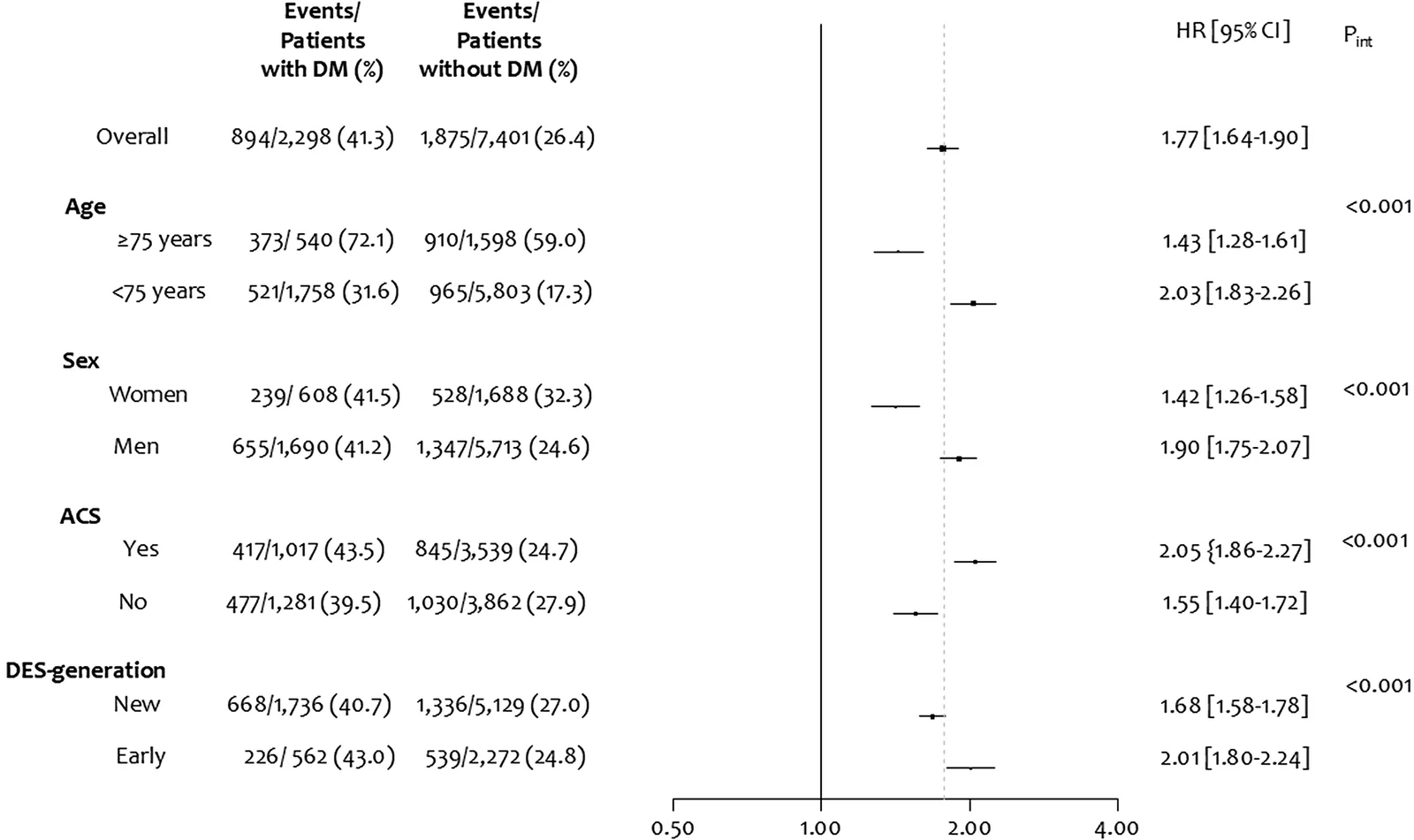
Interaction between DM and all-cause death after 10 years among subgroups of patients treated with PCI and DES implantation. The forest plot displays the association between DM and all-cause death out to 10 years among subgroups defined by age, sex, clinical presentation and DES-generation. Unadjusted hazard ratios (HRs) with 95% confidence intervals (CIs) are shown for patients with DM versus patients without DM. ACS= acute coronary syndrome; CI= confidence interval; DES= drug-eluting stent; DM= diabetes mellitus; PCI= percutaneous coronary intervention

Statistically significant interactions were found between DM and several outcomes of interest according to age, sex, clinical presentation and DES-generation (Fig. 2 and Supplemental Figs. 6–11). The subgroup analysis for the primary endpoint, all-cause death, revealed a significant interaction by age (P_interaction_ <0.001), with a more pronounced detrimental effect of DM in PCI patients aged < 75 years than in those aged ≥ 75 years. Similarly, there was evidence of significant interaction by sex (P_interaction_ <0.001), with a greater effect size observed in men than in women. The magnitude of the association between DM status and all-cause death changed significantly according to clinical presentation (P_interaction_ <0.001), with a stronger association with increased mortality for patients with DM presenting with ACS at the time of index PCI. Additionally, a significant interaction was observed according to the use of newer DES (P_interaction_ <0.001). Of note, despite significant differences in the magnitude of summary estimates for the primary endpoint, the direction of the association between DM status and all-cause death remained consistent across all subgroups.

## Discussion

This study presents a comprehensive assessment of the 10-year clinical impact of DM in nearly 10,000 patients undergoing PCI with DES in the setting of randomized trials. The large sample size and high long-term follow-up retention across all included trials strengthened the robustness of the 10-year analyses. The main findings of the present study can be summarized as follows:

i. DM was associated with a significantly increased risk of all-cause death across early, intermediate, and late follow-up. In addition, patients with DM had higher rates of definite ST and repeat revascularization compared to patients without DM.
ii. Despite the lack of an overall significant increase over the long term, DM was associated with a higher MI risk in the early post-PCI period, namely within the first month after index procedure.
iii. Patients with DM treated with insulin represent the highest risk phenotype among those treated with PCI and DES.

DM is associated with an accelerated atherosclerosis [19] and represents one of the most prevalent standard modifiable risk factors among patients undergoing PCI. Despite the lack of optimal control of these risk factors in PCI patients, poor glycemic control has been found to have the greatest impact on prognosis after percutaneous revascularization [20]. For this reason, investigating the long-term clinical impact of DM in patients undergoing PCI with DES is clinically relevant. The results of the present analysis suggest that DM negatively affects vascular processes, involving target and non-target vessels, leading to CAD progression and poor outcomes over a 10-year follow-up period. These events may reflect clinically recognized coronary disease progression or restenosis requiring repeat revascularization.

DM is an independent factor that contributes to the inferior efficacy of PCI treatment, even with the use of contemporary DES technologies [21, 22]. Previous studies have reported that mortality beyond 1 year after PCI is driven mainly by non-cardiac causes [23, 24]. In our study, however, cardiovascular death rates were higher than that of noncardiovascular death, although both categories contributed to the excess mortality compared with patients without DM. Interestingly, patients with DM experienced more definite ST and repeat revascularization involving both treated and untreated vessels over 10 years of follow-up. Notably, the risk of MI was significantly higher within the first year after PCI with DES, whereas no statistically significant association was detected thereafter. However, this finding should not be interpreted as evidence that there is no association between DM and MI risk in DES-treated patients after 1 year. On the one hand, this shows that disease progression involving both target and non-target vessels has a significant impact on late clinical outcomes [25, 26]. On the other hand, we cannot rule out the possibility of underreported MI events in the long term, which is common in patients with DM due to neuropathy. In fact, DM is associated with complications involving other vascular districts and adversely affects the microvasculature in multiple organs, leading to complications such as kidney failure and cognitive impairment [27]. These complications may explain the increased overall mortality observed in patients with DM in this analysis, which goes beyond CAD progression [5].

The landmark analyses indicate that the associations between DM and adverse outcomes after PCI were not consistent over time. The excess MI risk was predominantly observed within the first month, whereas the excess definite ST risk was detected between 30 days and 1 year. By contrast, rates of all-cause and cardiovascular death remained higher across all follow-up periods. The higher cumulative incidence of repeat revascularization, including nTVR, suggests that the long-term burden of adverse outcomes extends beyond the treated lesion to other coronary territories. These findings highlight the importance of distinguishing early ischemic and thrombotic events from long-term mortality and revascularization when describing prognosis after PCI. However, potential underestimation of MI and uncertainty in the later estimates limit interpretation of the temporal patterns.

Previous investigations of several catheter-based strategies have consistently reported that DM is a major determinant of increased mortality and device failure after percutaneous treatment for CAD [3]. The present study included PCI patients treated with both earlier and newer DES platforms: while newer DES platforms tended to reduce the local risk of adverse outcomes associated with DM, a significant residual excess risk remained, particularly in terms of mortality and repeat revascularization. This emphasizes that contemporary stent platforms alone are unlikely to be sufficient to fully offset the complex systemic consequences of DM [22, 28]. The temporal pattern of risk after PCI with DES observed in this analysis, with early amplification and persistent late divergence, supports the concept that atherosclerosis in patients with DM is a diffuse and progressive systemic process, that is only partly influenced by the local effect of specific DES platforms. Along the same line, this finding suggests that the mechanisms responsible for adverse outcomes in DM extend beyond device-related factors and include persistent systemic vascular inflammation, generalized endothelial dysfunction, and enhanced thrombotic susceptibility [19, 20, 29]. In this regard, the role of secondary prevention measures and aggressive control of other standard modifiable risk factors is as important as glycaemic management. Our findings should also be interpreted in the context of the treatment era in which the trials were conducted, before the introduction of sodium-glucose cotransporter 2 inhibitors (SGLT2i) and proprotein convertase subtilisin/kexin type 9 inhibitors (PCSK9i) into routine clinical practice. The observed event rates therefore provide an historical benchmark and may not fully reflect outcomes under contemporary medical therapy [30–32]. Cardiovascular outcomes trials of glucagon-like peptide-1 (GLP-1)​ receptor agonists provide an important context for these findings. In the Liraglutide Effect and Action in Diabetes: Evaluation of Cardiovascular Outcome Results (LEADER) trial, liraglutide reduced the primary composite of cardiovascular death, nonfatal MI, or nonfatal stroke compared with placebo in patients with type 2 diabetes at high cardiovascular risk, with a significant reduction in cardiovascular death. Similarly, the trial to Evaluate Cardiovascular and Other Long-term Outcomes with Semaglutide in Subjects with Type 2 Diabetes (SUSTAIN-6) reported fewer primary cardiovascular events with subcutaneous semaglutide than with placebo. More recently, the Semaglutide Cardiovascular Outcomes Trial (SOUL) demonstrated that oral semaglutide reduced the same composite cardiovascular endpoint in patients with type 2 DM and atherosclerotic cardiovascular disease, chronic kidney disease, or both. However, because the included trials largely preceded the widespread use of these therapies, our study cannot determine whether contemporary GLP-1 receptor agonists​ treatment would attenuate the observed differences between patients with and without DM treated with PCI and DES.

Moreover, patients with DM have an increased risk of heart failure​, particularly with preserved ejection fraction​ [33]. In fact, a comprehensive approach encompassing biomarker monitoring and management, lifestyle interventions, and pharmacological prevention has proven effective especially for those groups at the highest risk of adverse outcomes.

It is worth noting that insulin-treated patients displayed worse clinical outcomes after PCI with DES compared to patients not receiving insulin. However, there were no significant differences compared to patients with DM not receiving insulin in terms of MI and definite ST. The poor outcomes associated with insulin therapy in patients with DM may be due to more severe CAD, with longer-standing DM and greater comorbidity [34]. Indeed, while iatrogenic hyperinsulinemia is used to control hyperglycaemia in patients with DM, it may also promote proinflammatory macrophage responses and stimulate hormonal overactivation of signal transduction pathways being stimulated. This affects the progression of atherogenesis by disrupting the balanced synthesis and release of endothelial mediators, thereby disturbing haemodynamic control and cardiovascular function [35]. Indeed, the Future Revascularization Evaluation in Patients with Diabetes Mellitus: Optimal Management of Multivessel Disease (FREEDOM) trial, which compared coronary artery bypass grafting and PCI in patients with DM and multivessel CAD, revealed that the risk of adverse outcomes, including death, after coronary revascularization was high in insulin-treated patients, regardless of the revascularization strategy [36]. These findings suggest that insulin could serve as a marker for identifying high-risk patients, not only because of more severe insulin resistance and advanced CAD but also because of longer diabetes duration and extra-cardiac damage.

### Study limitations

Several limitations should be considered when interpreting the results of the current analysis. First, although we pooled the individual patient data from five randomized clinical trials to increase the overall statistical power and investigate the clinical outcomes of patients with DM after PCI with DES, each trial was not specifically powered to compare clinical outcomes in the subgroup of patients with or without DM. Second, despite statistical adjustment for relevant factor variables, we cannot exclude the impact of residual confounders on overall estimations. Although the source trials randomized DES allocation, DM status was not part of the randomization process. The present comparisons are observational and should be interpreted as associations, without inferring causal effects of DM or benefits from specific surveillance, pharmacological, or interventional strategies. Third, information on glycemic control, duration of DM and secondary organ damage was unavailable. Similarly, we cannot exclude possible changes in therapy for glycemic control and other secondary prevention measures over time. Fourth, the studies included in this pooled analysis were conducted over 10 years ago, so the prescribed therapies may no longer represent the current standard of care. Fifth, although the events were defined consistently, they were adjudicated within the individual studies rather than by a single committee, which could introduce heterogeneity in event reporting. Finally, the proportional hazards assumption was not fulfilled for some endpoints, indicating time-varying effects. While landmark analyses were performed to explore temporal patterns of risk, the reported overall risk estimates should be interpreted as time-averaged.

## Conclusions

DM was associated with distinct temporal patterns of adverse outcomes after PCI with DES. The excess MI risk was predominantly observed within the first month, and the excess definite ST risk between 30 days and 1 year, whereas mortality remained higher across follow-up intervals and repeat revascularization was more frequent over 10 years. Despite advances in stent design technology, patients with DM undergoing PCI with DES, have a markedly inferior long-term prognosis compared with patients without DM. These results further emphasize the need for intensified secondary prevention and bespoke interventional and pharmacological strategies for this patient group, while providing a long-term benchmark against which novel management approaches should be evaluated in patients with DM undergoing PCI.

## Supplementary Information

Below is the link to the electronic supplementary material.

Supplementary Material 1

## Data Availability

The data are, however, available upon reasonable request and with the permission of the principal investigators of each trial included in the DECADE cooperation.

## Abbreviations

CAD: Coronary artery disease
DES: Drug-eluting stent
MI: Myocardial infarction
nTVR: non-target vessel revascularization
PCI: Percutaneous coronary intervention
ST: Stent thrombosis
TLR: Target lesion revascularization
TVR: Target vessel revascularization
