# Supplementary material for: Diabetes mellitus and 10-year clinical outcomes after percutaneous coronary intervention with drug-eluting stents: insights from the DECADE cooperation
Source: Cardiovasc Diabetol. 2026 Nov 11;25:332. doi: 10.1186/s12933-026-03434-3 (PMC13761022; doi:10.1186/s12933-026-03434-3)
Supplement: Supplementary file 1 [file 12933_2026_3434_MOESM1_ESM.docx]

**Supplemental Material**

[**SUPPLEMENTAL FIGURES LEGEND**	3](#_Toc243233486)

[**SUPPLEMENTAL TABLES**	7](#_Toc243233490)

[**Supplemental Table 1. Outcome definitions in DECADE cooperation trials.**	7](#_Toc243233491)

[**Supplemental Table 3. Trial-specific median follow-up duration and number and proportion of patients lost to follow-up at 1, 5, and 10 years, according to DM diagnosis at baseline.**	13](#_Toc243233493)

[**Supplemental Table 4. Procedural and angiographic characteristics at lesion level as per DM diagnosis at baseline**	14](#_Toc243233494)

[**Supplemental Table 5. Ten-year clinical outcomes as per DM diagnosis at baseline**	15](#_Toc243233495)

[**Supplemental Table 6. Sensitivity analysis of clinical outcomes as per DM diagnosis at baseline using expanded multivariable adjustment and imputed missing covariate data**	16](#_Toc243233496)

[**Supplemental Table 7. Results of Schoenfelds global goodness-of-fit test for outcomes of interest**	17](#_Toc243233497)

[**Supplemental Table 8. Trial-specific clinical outcomes and hazard ratios as per DM diagnosis at baseline in EXAMINATION.**	18](#_Toc243233498)

[**Supplemental Table 9. Trial-specific clinical outcomes and hazard ratios as per DM diagnosis at baseline in ISAR-TEST 4.**	19](#_Toc243233499)

[**Supplemental Table 10. Trial-specific clinical outcomes and hazard ratios as per DM diagnosis at baseline in ISAR-TEST 5.**	20](#_Toc243233500)

[**Supplemental Table 11. Trial-specific clinical outcomes and hazard ratios as per DM diagnosis at baseline in SIRTAX.**	21](#_Toc243233501)

[**Supplemental Table 12. Trial-specific clinical outcomes and hazard ratios as per DM diagnosis at baseline in SORT OUT III.**	22](#_Toc243233502)

[**Supplemental Table 13. Clinical characteristics of patients with DM as per insulin treatment at baseline**	23](#_Toc243233503)

[**Supplemental Table 14. Ten-year clinical outcomes according to insulin therapy among patients with DM diagnosis at baseline.**	24](#_Toc243233504)

[**SUPPLEMENTAL FIGURES**	25](#_Toc243233505)

[**Supplemental Figure 1. Ten-Year Cumulative Incidence of TLR (A), TVR (B), nTVR (C) according to DM diagnosis at baseline.**	25](#_Toc243233506)

[**Supplemental Figure 2. Landmark analysis for all-cause death (A), CV-death (B), MI (C), definite ST (D) according to DM diagnosis at baseline.**	26](#_Toc243233507)

[**Supplemental Figure 3. Landmark Analysis for myocardial infarction according to DM diagnosis at baseline.**	26](#_Toc243233508)

[**Supplemental Figure 4. Landmark Analysis for definite ST according to DM diagnosis at baseline**	27](#_Toc243233509)

[**Supplemental Figure 5. Trial-specific and pooled estimates of the association between DM diagnosis at baseline and all-cause death over 10 years.**	27](#_Toc243233510)

[**Supplemental Figure 6. Interaction between DM and cardiovascular death after 10 years among subgroups of patients treated with PCI and DES implantation.**	28](#_Toc243233511)

[**Supplemental Figure 7. Interaction between DM and myocardial infarction after 10 years among subgroups of patients treated with PCI and DES implantation.**	29](#_Toc243233512)

[**Supplemental Figure 8. Interaction between DM and stent thrombosis after 10 years among subgroups of patients treated with PCI and DES implantation.**	30](#_Toc243233513)

[**Supplemental Figure 9. Interaction between DM and target lesion revascularization after 10 years among subgroups of patients treated with PCI and DES implantation**	31](#_Toc243233514)

[**Supplemental Figure 10. Interaction between DM and target vessel revascularization after 10 years among subgroups of patients treated with PCI and DES implantation.**	32](#_Toc243233515)

[**Supplemental Figure 11. Interaction between DM and non- target vessel revascularization after 10 years among subgroups of patients treated with PCI and DES implantation.**	33](#_Toc243233516)

# **SUPPLEMENTAL FIGURES LEGEND**

**Supplemental Figure 1.** **Ten-Year Cumulative Incidence of TLR (A), TVR (B), nTVR (C) according to DM diagnosis at baseline.**

Cumulative incidence function curves and subdistribution adjusted hazard ratio (HRadj) with accompanying 95% confidence interval (CI) for TLR (A), TVR (B), nTVR (C) as per diabetic status at baseline. Adjusted curves are derived from Cox/Fine and Gray model. nTVR=non target vessel revascularization; TLR= target lesion revascularization; TVR= target vessel revascularization

**Supplemental Figure 2. Landmark Analysis for All-cause death (A), CV- death (B), myocardial infarction (C), definite ST (D) according to DM diagnosis at baseline.**

Cumulative incidence function curves and subdistribution adjusted hazard ratio (HR_adj_) with accompanying 95% confidence interval (CI) for All-cause death (A), CV- death (B), myocardial infarction (C), definite ST (D) as per diabetic status at baseline according to the landmark analysis (0 to 1 year, 1 to 5 years, 5 to 10 years). Adjusted curves are derived from Cox/Fine and Gray models. CV= cardiovascular; ST= stent thrombosis

**Supplemental Figure 3. Landmark Analysis for myocardial infarction according to DM diagnosis at baseline**.

Cumulative incidence function curves and subdistribution hazard ratio (HR_adj_) with accompanying 95% CI myocardial infarction as per diabetic status at baseline according to the landmark analysis (0 to 1 month, 1 to 12 months). Adjusted curves are derived from Cox/Fine and Gray models.

**Supplemental Figure 4. Landmark Analysis for definite ST according to DM diagnosis at baseline.**

Cumulative incidence function curves and subdistribution hazard ratio (HR_adj_) with accompanying 95% CI definite ST as per diabetic status at baseline according to the landmark analysis (0 to 1 month, 1 to 12 months). Adjusted curves are derived from Cox/Fine and Gray models. ST= stent thrombosis

**Supplemental Figure 5. Trial-specific and pooled estimates of the association between diabetes mellitus and 10-year all-cause mortality.**

Forest plot showing unadjusted trial-specific hazard ratios (HRs) and 95% confidence intervals (CIs) for patients with versus without diabetes mellitus. Squares represent individual trial estimates, with their size proportional to the common-effect model weights; horizontal lines indicate 95% CIs. Diamonds represent pooled estimates from common-effect and random-effects inverse-variance meta-analyses. Between-trial heterogeneity was assessed using Cochran’s Q test and quantified using I² and τ².

**Supplemental Figure 6. Interaction between DM and cardiovascular death after 10 years among subgroups of patients treated with PCI and DES implantation.**

Forest plot showing the association between diabetes mellitus and cardiovascular death through 10 years of follow-up across prespecified subgroups. Hazard ratios (HRs) with 95% confidence intervals (CIs) are shown for diabetic versus nondiabetic patients. ACS = acute coronary syndrome; CI = confidence interval; DES = drug-eluting stent; HR = hazard ratio.

**Supplemental Figure 7. Interaction between DM and myocardial infarction after 10 years among subgroups of patients treated with PCI and DES implantation.**

Forest plot showing the association between diabetes mellitus and myocardial infarction through 10 years of follow-up across prespecified subgroups. Hazard ratios (HRs) with 95% confidence intervals (CIs) are shown for diabetic versus nondiabetic patients. ACS = acute coronary syndrome; CI = confidence interval; DES = drug-eluting stent; HR = hazard ratio.

**Supplemental Figure 8. Interaction between DM and definite stent thrombosis after 10 years among subgroups of patients treated with PCI and DES implantation.**

Forest plot showing the association between diabetes mellitus and stent thrombosis through 10 years of follow-up across prespecified subgroups. Hazard ratios (HRs) with 95% confidence intervals (CIs) are shown for diabetic versus nondiabetic patients. ACS = acute coronary syndrome; CI = confidence interval; DES = drug-eluting stent; HR = hazard ratio.

**Supplemental Figure 9. Interaction between DM and target lesion revascularization after 10 years among subgroups of patients treated with PCI and DES implantation.**

Forest plot showing the association between diabetes mellitus and target lesion revascularization through 10 years of follow-up across prespecified subgroups. Hazard ratios (HRs) with 95% confidence intervals (CIs) are shown for diabetic versus nondiabetic patients. ACS = acute coronary syndrome; CI = confidence interval; DES = drug-eluting stent; HR = hazard ratio.

**Supplemental Figure 10. Interaction between DM and target vessel revascularization after 10 years among subgroups of patients treated with PCI and DES implantation.**

Forest plot showing the association between diabetes mellitus and target vessel revascularization through 10 years of follow-up across prespecified subgroups. Hazard ratios (HRs) with 95% confidence intervals (CIs) are shown for diabetic versus nondiabetic patients. ACS = acute coronary syndrome; CI = confidence interval; DES = drug-eluting stent; HR = hazard ratio.

**Supplemental Figure 11. Interaction between DM and non-target vessel revascularization after 10 years among subgroups of patients treated with PCI and DES implantation.**

Forest plot showing the association between diabetes mellitus and non-target vessel revascularization through 10 years of follow-up across prespecified subgroups. Hazard ratios (HRs) with 95% confidence intervals (CIs) are shown for diabetic versus nondiabetic patients. ACS = acute coronary syndrome; CI = confidence interval; DES = drug-eluting stent; HR = hazard ratio.

# **SUPPLEMENTAL TABLES**

# **Supplemental Table 1. Outcome definitions in DECADE cooperation trials.**

| **Trial**  **(enrollment period)** | **Cardiovascular death** | **MI** | **Definite ST** | **TLR** | **TVR** | **nTVR** |
| --- | --- | --- | --- | --- | --- | --- |
| **EXAMINATION (2008-2010)** | Any death due to proximate cardiac cause (eg, MI, low-output failure, fatal arrhythmia), unwitnessed death and death of unknown cause, and all procedure-related deaths, including those related to concomitant treatment, will be classified as cardiac death. | Recurrent myocardial infarction is defined according to the WHO extended definition. Both periprocedural and spontaneous MI were assessed. For both situations a dedicated algorithm was used in the adjudication process (also adopted from the WHO extended definition). | Academic Research Consortium criteria | TLR is defined as any repeat percutaneous intervention of the target lesion or bypass surgery of the target vessel performed for restenosis or other complication of the target lesion. | TVR is defined as any repeat percutaneous intervention or surgical bypass of any segment of the target vessel. The target vessel is defined as the entire major coronary vessel proximal and distal to the target lesion, which includes upstream and downstream branches and the target lesion itself. | NR |
| **ISAR TEST 4 (2007-2008)** | Death due to any of the following: acute MI; cardiac perforation/pericardial tamponade; arrhythmia or conduction abnormality; stroke within 30 days of the procedure or stroke suspected of being related to the procedure; death due to complication of the procedure, including bleeding, vascular repair, transfusion reaction, or bypass surgery; or any death in which a cardiac cause cannot be excluded | MI related to procedure was defined as either an increase in CK-MB (or CK) ≥3 upper limit of normal (ULN) and at least 50% over the most recent pre- PCI levels, or the development of new ECG changes consistent with MI and CK-MB (CK) elevation higher than the ULN at two measurements for patients undergoing DES implantation in setting of stable angina pectoris or non-ST-segment elevation acute coronary syndrome (NSTE-ACS) and falling or normal CK-MB (CK) levels. Recurrent chest pain lasting >30 min with either new ECG changes consistent with second MI or next CK-MB (CK) level at least 8–12 h after PCI elevated at least 50% above the previous level was considered procedure-related MI for patients presenting with NSTE-ACS and elevated CK-MB (CK) level prior to PCI. Spontaneous MI was defined as any CK-MB increase with or without the development of Q-waves on ECG. | Academic Research Consortium criteria | Target lesion revascularization was defined as any ischemia-driven repeat percutaneous coronary intervention of the target lesion or bypass surgery of the target vessel. | Any ischaemia-driven repeat PCI or bypass surgery revascularization of any segment of the treated coronary vessel proximal or distal to the treated segment and including upstream and downstream side branch vessels. | NR |
| **ISAR TEST 5 (2008-2009)** | Death due to any of the following: acute MI; cardiac perforation/pericardial tamponade; arrhythmia or conduction abnormality; stroke within 30 days of the procedure or stroke suspected of being related to the procedure; death due to complication of the procedure, including bleeding, vascular repair, transfusion reaction, or bypass surgery; or any death in which a cardiac cause cannot be excluded. | Spontaneous MI was defined as any creatine kinase MB or troponin increase with or without the development of Q waves on ECG. | Academic Research Consortium criteria | Target lesion revascularization was defined as any ischemia-driven repeat percutaneous coronary intervention of the target lesion or bypass surgery of the target vessel. | NR | NR |
| **SIRTAX (2003-2004)** | NR | The diagnosis of MI was based on the presence of new Q waves in at least two contiguous leads and an elevated creatine kinase MB fraction. In the absence of pathologic Q waves, the diagnosis of MI was based on an increase in the creatine kinase level to more than twice the upper limit of the normal range with an elevated level of creatine kinase MB or troponin I | Acute coronary syndrome with angiographic documentation of either occlusion of the target lesion or thrombus within the previously stented segment. | Target-lesion revascularization was defined as revascularization for a stenosis within the stent or within the 5-mm borders adjacent to the stent. | Revascularization was considered to be driven by ischemia if the visually estimated stenosis of any target lesion or vessel was at least 50 percent of the diameter of the target lesion and vessel in the presence of ischemic signs or symptoms, or if the stenosis was at least 70 percent of the diameter of the vessel even in the absence of ischemic signs or symptoms. | NR |
| **SORT OUT III (2006-2007)** | Any death due to an evident cardiac cause, death related to percutaneous coronary intervention, unwitnessed death, and death from unknown causes. | MI was defined in accordance with the Joint ESC/ACCF/AHA/WHF Task Force for the Redefinition of MI but procedure-related MI was excluded as an endpoint. | Academic Research Consortium criteria | Repeat revascularization caused by stenosis within the stent or within a 5 mm border proximal or distal to the stent. | Any repeat percutaneous coronary intervention or surgical bypass of any segment within the entire major coronary vessel that was proximal and distal to a target lesion, including upstream and downstream branches, and the target lesion itself. | NR |

## **Supplemental Table 2. Proportion of participants and DES platforms as per DM diagnosis at baseline as reported among trials included in the DECADE cooperation.**

| Characteristics | Patients  with DM (n= 2,298) | Patients  without DM (n= 7,401) | *P* value |
| --- | --- | --- | --- |
| Trial, n (%) |  |  | <0.001 |
| *EXAMINATION* | 137 (6.0) | 613 (8.3) |  |
| *ISAR-TEST 4* | 753 (32.8) | 1,850 (25.0) |  |
| *ISAR-TEST 5* | 870 (37.9) | 2,132 (28.8) |  |
| *SIRTAX* | 201 (8.8) | 811 (11.0) |  |
| *SORT-OUT III* | 337 (14.7) | 1,995 (27.0) |  |
| DES type, n (%) |  |  | <0.001 |
| *Yukon Choice BP-SES* | 376 (16.4) | 923 (12.5) |  |
| *Cypher PP-SES* | 469 (20.4) | 1,856 (25.1) |  |
| *Endeavor PP-ZES* | 169 (7.4) | 993 (13.4) |  |
| *ISAR VIVO/*  *Coroflex PF-SPES* | 575 (25.0) | 1,427 (19.3) |  |
| *Resolute PP-ZES* | 295 (12.8) | 705 (9.5) |  |
| *Taxus PP-PES* | 93 (4.1) | 416 (5.6) |  |
| *Xience PP-EES* | 321 (14.0) | 1,081 (14.6) |  |
| DES generation, n (%) |  |  | <0.001 |
| *Early* | 562 (24.5) | 2,272 (30.7) |  |
| *New* | 1,736 (75.5) | 5,129 (69.3) |  |

Data are shown as counts (percentages). Data were analyzed at a patient-level. Trial acronyms: EXAMINATION: Clinical Evaluation of the Xience-V Stent in Acute Myocardial Infarction; ISAR-TEST 4: Intracoronary Stenting and Angiographic Results: Test Efficacy of 3 Limus-Eluting Stents; ISAR-TEST 5: Intracoronary Stenting and Angiographic Results: Test Efficacy of Sirolimus- and Probucol-Eluting Versus Zotarolimus-Eluting Stents; SIRTAX: Sirolimus-Eluting Versus Paclitaxel-Eluting Stents for Coronary Revascularization; SORT OUT III: Randomized Clinical Comparison of the Endeavor and the Cypher Coronary Stents in Non-Selected Angina Pectoris Patients. BP: biodegradable polymer; DES: drug-eluting stent; PF: polymer free; PP: permanent polymer; PES: paclitaxel-eluting stent; SES: sirolimus-eluting stent; SPES: sirolimus- and probucol-eluting stent; ZES: zotarolimus-eluting stent. Early-generation DES included Cypher sirolimus-eluting and Taxus paclitaxel-eluting stents. New-generation DES included Yukon Choice, Endeavor, ISAR VIVID/Coroflex, Resolute, and Xience stent.

## **Supplemental Table 3. Trial-specific median follow-up duration and number and proportion of patients lost to follow-up at 1, 5, and 10 years, according to DM diagnosis at baseline.**

| **Trial** |  | **Patients**  **with DM** | **Patients**  **without DM** |
| --- | --- | --- | --- |
| **EXAMINATION** |  | | |
|  | Median Follow-up, years | 9.86 | 9.86 |
|  | Lost at 1 year, n(%) | 0 (0.0) | 0 (0.0) |
|  | Lost at 5 years, n(%) | 11 (8.66) | 23 (3.85) |
|  | Lost at 10 years, n (%) | 33 (26.0) | 108 (18.1) |
| **ISAR TEST 4** |  | | |
|  | Median Follow-up, years | 11.01 | 11.25 |
|  | Lost at 1 year, n(%) | 20 (2.85) | 15 (0.84) |
|  | Lost at 5 years, n(%) | 139 (19.8) | 238 (13.4) |
|  | Lost at 10 years, n (%) | 349 (49.8) | 602 (33.8) |
| **ISAR TEST 5** |  | | |
|  | Median Follow-up, years | 10.3 | 9.97 |
|  | Lost at 1 year, n(%) | 0 (0.0) | 1 (0.05) |
|  | Lost at 5 years, n(%) | 161 (19.7) | 238 (11.5) |
|  | Lost at 10 years, n (%) | 351 (43.0) | 600 (29.1) |
| **SIRTAX** |  | | |
|  | Median Follow-up, years | 10.47 | 10.43 |
|  | Lost at 1 year, n(%) | 0 (0.0) | 2 (0.25) |
|  | Lost at 5 years, n(%) | 34 (17.7) | 54 (6.80) |
|  | Lost at 10 years, n (%) | 78 (40.6) | 207 (26.1) |
| **SORT OUT III** |  | | |
|  | Median Follow-up, years | 10.0 | 10.0 |
|  | Lost at 1 year, n(%) | 0 (0.0) | 2 (0.10) |
|  | Lost at 5 years, n(%) | 38 (11.9) | 184 (9.40) |
|  | Lost at 10 years, n (%) | 97 (30.3) | 441 (22.5) |

### **Supplemental Table 4. Procedural and angiographic characteristics at lesion level as per DM diagnosis at baseline**

| Characteristics | Number of analyzed lesion in  patients  with DM  (n= 3,145) | Number of analyzed lesion in patients without DM  (n= 10,007) | *P* value |
| --- | --- | --- | --- |
| Target vessel, n (%) |  |  | 0.063 |
| *Left anterior descending coronary artery* | 1,333 (42.4) | 4,454 (44.5) |  |
| *Left circumflex coronary artery* | 827 (26.3) | 2,394 (23.9) |  |
| *Left main coronary artery* | 15 (0.48) | 63 (0.63) |  |
| *Right coronary artery* | 959 (30.5) | 3,059 (30.6) |  |
| *Saphenous vein graft* | 11 (0.35) | 37 (0.37) |  |
| Bifurcation involved and treated, n (%) | 558 (21.9) | 1,587 (23.9) | 0.046 |
| ACC/AHA classification of target lesion, n (%) |  |  | <0.001 |
| *A/B1* | 995 (33.2) | 3,544 (37.9) |  |
| *B2/C* | 2,005 (66.8) | 5,799 (62.1) |  |
|  | **(n= 3,139)** | **(n= 9,973)** |  |
| Pre-procedural reference vessel diameter, mm | 2.74 (2.42-3.05) | 2.80 (2.46-3.12) | <0.001 |
| Pre-procedural minimal lumen diameter, mm | 0.88 (0.59-1.20) | 0.86 (0.54-1.20) | 0.060 |
| Balloon diameter, mm | 3.00 (2.64-3.49) | 3.04 (2.75-3.50) | <0.001 |
| Maximal balloon pressure, atm | 16.0 (13.0-18.0) | 15.0 (12.2-17.2) | <0.001 |
| Total stented length, mm | 23.0 (18.0-30.0) | 18.0 (15.0-28.0) | <0.001 |
| Number of stents, n | 1.00 (1.00-2.00) | 1.00 (1.00-2.00) | <0.001 |
| Post-procedural minimal lumen diameter | 2.51 (2.20-2.83) | 2.57 (2.25-2.90) | <0.001 |
| Post-procedural diameter stenosis, % | 11.0 (7.66-15.1) | 10.7 (7.12-14.8) | 0.002 |

Data are shown as median (interquartile range) or n (%). Data were analyzed at a lesion-level.

## **Supplemental Table 5. Ten-year clinical outcomes as per DM diagnosis at baseline**

| Endpoint | sHR [95% CI] | *P* value | sHR_adj_ [95% CI] | *P* value |
| --- | --- | --- | --- | --- |
| All-cause death | - | - | - | - |
| Cardiovascular death | 1.97 [1.77-2.19] | <0.001 | 1.59 [1.45-1.76] | <0.001 |
| Non-cardiovascular death | 1.36 [1.21-1.53] | <0.001 | 1.44 [1.23-1.68] | <0.001 |
| Myocardial infarction | 1.19 [1.01-1.39] | 0.037 | 1.23 [0.87-1.73] | 0.235 |
| Definite ST | 1.22 [0.86-1.73] | 0.260 | 1.47 [1.06-2.06] | 0.022 |
| Target lesion revascularization | 1.43 [1.28-1.60] | <0.001 | 1.28 [1.14-1.42] | <0.001 |
| Target vessel revascularization | 1.39 [1.25-1.53] | <0.001 | 1.21 [1.09-1.35] | <0.001 |
| Non-target vessel revascularization | 1.52 [1.38-1.68] | <0.001 | 1.15 [1.02-1.30] | 0.021 |

Unadjusted and adjusted subdistribution hazard ratios (sHRs), with corresponding 95% confidence intervals (CIs), were estimated using Fine-Gray regression models. Fine-Gray estimates are not applicable to all-cause death. Multivariable models were adjusted for age, body mass index, sex, smoking status, DES generation, hypertension, hypercholesterolemia, prior myocardial infarction, acute coronary syndrome presentation, multivessel disease, and treatment of the left anterior descending coronary artery, with robust standard errors with clustering for trial. sHR: subdistribution hazard ratio; sHRadj: adjusted subdistribution hazard ratio; CI: confidence interval; DM: diabetes mellitus; DES: drug-eluting stent; ST: stent thrombosis.

## **Supplemental Table 6. Sensitivity analysis of clinical outcomes as per DM diagnosis at baseline using expanded multivariable adjustment and imputed missing covariate data**

| Endpoint | aHR [95% CI] | *P* value | sHR_adj_ [95% CI] | *P* value |
| --- | --- | --- | --- | --- |
| All-cause death, n(%) | 1.58 [1.46-1.72] | <0.001 | - | - |
| Cardiovascular death, n(%) | 1.73 [1.55-1.94] | <0.001 | 1.61 [1.43-1.81] | <0.001 |
| Non-cardiovascular death, n(%) | 1.43 [1.27-1.62] | <0.001 | 1.31 [1.16-1.48] | <0.001 |
| Myocardial infarction, n(%) | 1.30 [1.10-1.54] | 0.002 | 1.23 [1.03-1.45] | 0.018 |
| Definite ST, n(%) | 1.59 [1.14-2.21] | 0.006 | 1.44 [1.00-2.06] | 0.044 |
| Target lesion revascularization n(%) | 1.44 [1.27-1.62] | <0.001 | 1.37 [1.21-1.54] | <0.001 |
| Target vessel revascularization, n(%) | 1.35 [1.21-1.50] | <0.001 | 1.28 [1.16-1.44] | <0.001 |
| Non-target vessel revascularization, n(%) | 1.37 [1.23-1.52] | <0.001 | 1.29 [1.16-1.44] | <0.001 |

Multivariable models were adjusted for age, body mass index, sex, smoking status, DES generation, hypertension, hypercholesterolemia, prior myocardial infarction, acute coronary syndrome presentation, multivessel disease, treatment of the left anterior descending coronary artery, left ventricular ejection fraction, ACC/AHA lesion classification, preprocedural reference vessel diameter, preprocedural percentage diameter stenosis, and total stented length, with robust standard errors clustered by trial. HR: hazard ratio; aHRadj: hazard ratio; ST: stent thrombosis; CI: confidence interval; ACC/AHA: American College of Cardiology/American Heart Association.

## **Supplemental Table 7. Results of Schoenfelds global goodness-of-fit test for outcomes of interest**

| Endpoint | *P* value |
| --- | --- |
| All-cause death | 0.032 |
| Cardiovascular death | 0.052 |
| Myocardial infarction | 0.001 |
| Definite ST | 0.029 |
| Target lesion revascularization | 0.195 |
| Target vessel revascularization | 0.205 |
| Non-target vessel revascularization | 0.103 |

ST: stent thrombosis;

## **Supplemental Table 8. Trial-specific clinical outcomes and hazard ratios as per DM diagnosis at baseline in EXAMINATION.**

| Endpoint | HR [95% CI] | *P* value | sHR [95% CI] | *P* value |
| --- | --- | --- | --- | --- |
| All-cause death | 1.89 [1.31-2.75] | <0.001 | - | - |
| Cardiovascular death | 2.10 [1.32-2.19] | 0.002 | 2.08 [1.31-3.30] | 0.002 |
| Non-cardiovascular death | 1.85 [0.85-2.94] | 0.147 | 1.43 [0.77-2.66] | 0.254 |
| Myocardial infarction | 1.65 [0.81-3.38] | 0.168 | 1.52 [0.74-3.10] | 0.253 |
| Definite ST | 1.60 [0.52-4.94] | 0.413 | 1.50 [0.48-4.66] | 0.479 |
| Target lesion revascularization | 1.14 [0.53-2.46] | 0.739 | 1.06 [0.49-2.30] | 0.876 |
| Target vessel revascularization | 1.43 [0.81-2.49] | 0.208 | 1.32 [0.76-2.31] | 0.323 |
| Non-target vessel revascularization | 1.49 [0.78-2.86] | 0.114 | 1.41 [0.74-2.70] | 0.300 |

HR: hazard ratio; ST: stent thrombosis; CI: confidence interval. Hazard ratios and 95% CIs are derived from cox proportional hazard models and Fine-Gray regression models. Fine-Gray estimates are not applicable to all-cause death.

## **Supplemental Table 9. Trial-specific clinical outcomes and hazard ratios as per DM diagnosis at baseline in ISAR-TEST 4.**

| Endpoint | HR [95% CI] | *P* value | sHR [95% CI] | *P* value |
| --- | --- | --- | --- | --- |
| All-cause death | 1.68 [1.45-1.94] | <0.001 | - | - |
| Cardiovascular death | 1.71 [1.40-2.09] | <0.001 | 1.59 [1.32-1.93] | <0.001 |
| Non-cardiovascular death | 1.63 [1.32-2.03] | <0.001 | 1.57 [1.26-1.94] | <0.001 |
| Myocardial infarction | 1.05 [0.76-1.45] | 0.766 | 1.00 [0.72-1.38] | 0.974 |
| Definite ST | 2.16 [1.06-4.35] | 0.032 | 2.08 [1.03-4.21] | 0.041 |
| Target lesion revascularization | 1.32 [1.09-1.61] | 0.004 | 1.25 [1.04-1.52] | 0.020 |
| Target vessel revascularization | 1.28 [1.07-1.54] | 0.005 | 1.24 [1.04-1.48] | 0.016 |
| Non-target vessel revascularization | 1.12 [0.95-1.32] | 0.176 | 1.04 [0.89-1.23] | 0.580 |

HR: hazard ratio; ST: stent thrombosis; CI: confidence interval. Hazard ratios and 95% CIs are derived from cox proportional hazard models and Fine-Gray regression models. Fine-Gray estimates are not applicable to all-cause death.

## **Supplemental Table 10. Trial-specific clinical outcomes and hazard ratios as per DM diagnosis at baseline in ISAR-TEST 5.**

| Endpoint | HR [95% CI] | *P* value | sHR [95% CI] | *P* value |
| --- | --- | --- | --- | --- |
| All-cause death | 1.64 [1.45-1.87] | <0.001 | - | - |
| Cardiovascular death | 1.81 [1.55-2.12] | <0.001 | 1.73 [1.50-2.03] | <0.001 |
| Non-cardiovascular death | 1.35 [1.07-1.69] | 0.011 | 1.25 [1.00-1.58] | 0.048 |
| Myocardial infarction | 1.83 [1.33-2.52] | <0.001 | 1.76 [1.28-2.43] | <0.001 |
| Definite ST | 2.58 [1.13-5.88] | 0.024 | 2.47 [1.07-5.69] | 0.033 |
| Target lesion revascularization | 1.48 [1.24-1.77] | <0.001 | 1.38 [1.16-1.65] | <0.001 |
| Target vessel revascularization | 1.40 [1.20-1.64] | <0.001 | 1.33 [1.14-1.55] | <0.001 |
| Non-target vessel revascularization | 1.39 [1.22-1.60] | <0.001 | 1.31 [1.15-1.50] | <0.001 |

HR: hazard ratio; ST: stent thrombosis; CI: confidence interval. Hazard ratios and 95% CIs are derived from cox proportional hazard models and Fine-Gray regression models. Fine-Gray estimates are not applicable to all-cause death.

## **Supplemental Table 11. Trial-specific clinical outcomes and hazard ratios as per DM diagnosis at baseline in SIRTAX.**

| Endpoint | HR [95% CI] | *P* value | sHR [95% CI] | *P* value |
| --- | --- | --- | --- | --- |
| All-cause death | 2.33 [1.76-3.07] | <0.001 | - | - |
| Cardiovascular death | 2.12 [1.73-4.06] | <0.001 | 2.42 [1.59-3.70] | <0.001 |
| Non-cardiovascular death | 2.65 [1.73-4.06] | <0.001 | 2.42 [1.59-3.70] | <0.001 |
| Myocardial infarction | 0.98 [0.57-1.68] | 0.937 | 0.90 [0.52-1.55] | 0.709 |
| Definite ST | 0.90 [0.43-1.84] | 0.773 | 0.84 [0.41-1.72] | 0.639 |
| Target lesion revascularization | 0.91 [0.63-1.33] | 0.640 | 0.84 [0.58-1.23] | 0.377 |
| Target vessel revascularization | 0.95 [0.68-1.32] | 0.770 | 0.87 [0.63-1.21] | 0.428 |
| Non-target vessel revascularization | 1.51 [1.07-2.12] | 0.017 | 1.35 [0.97-1.89] | 0.072 |

HR: hazard ratio; ST: stent thrombosis; CI: confidence interval. Hazard ratios and 95% CIs are derived from cox proportional hazard models and Fine-Gray regression models. Fine-Gray estimates are not applicable to all-cause death.

## **Supplemental Table 12. Trial-specific clinical outcomes and hazard ratios as per DM diagnosis at baseline in SORT OUT III.**

| Endpoint | HR [95% CI] | *P* value | sHR [95% CI] | *P* value |
| --- | --- | --- | --- | --- |
| All-cause death | 1.57 [1.29-1.90] | <0.001 | - | - |
| Cardiovascular death | 1.84 [1.21-2.80] | 0.005 | 1.78 [1.17-2.72] | 0.006 |
| Non-cardiovascular death | 1.50 [1.21-1.87] | <0.001 | 1.45 [1.17-1.80] | <0.001 |
| Myocardial infarction | 1.59 [1.18-2.14] | 0.002 | 1.49 [1.11-1.98] | 0.007 |
| Definite ST | 1.10 [0.46-2.64] | 0.817 | 1.07 [0.45-2.56] | 0.870 |
| Target lesion revascularization | 1.69 [1.15-2.46] | 0.007 | 1.62 [1.11-2.37] | 0.012 |
| Target vessel revascularization | 1.50 [1.09-2.06] | 0.012 | 1.44 [1.05-1.98] | 0.023 |
| Non-target vessel revascularization | - | - | - | - |

HR: hazard ratio; ST: stent thrombosis; CI: confidence interval. Hazard ratios and 95% CIs are derived from cox proportional hazard models and Fine-Gray regression models. Fine-Gray estimates are not applicable to all-cause death.

# **Supplemental Table 13. Clinical characteristics of patients with DM as per insulin treatment at baseline**

| Characteristics | Patients  Insulin treated  (n= 635) | Patients  Noninsulin treated  (n= 1663) | *P* value |
| --- | --- | --- | --- |
| Sex, n (%) |  |  | 0.030 |
| *Female* | 189 (29.8) | 419 (25.2) |  |
| Smoker | 108 (17.1) | 330 (20.1) | 0.114 |
| Hypertension, n (%) | 459 (73.2) | 1238 (74.8) | 0.229 |
| Hypercholesterolemia, n (%) | 436 (68.7) | 1103 (66.6) | 0.384 |
| Previous MI (%) | 197 (31.1) | 462 (27.9) | 0.148 |
| Clinical presentation, n (%) |  |  | 0.002 |
| *Stable Angina* | 341 (53.7) | 940 (56.5) |  |
| *Unstable Angina* | 138 (21.7) | 259 (15.6) |  |
| *Acute MI* | 156 (24.6) | 464 (27.9) |  |
| *Total ACS, n (%)* | 294 (46.3) | 723 (43.5) | 0.241 |
| Number of lesions | 1.0 (1.0 - 2.0) | 1.0 (1.0 - 2.0) | 0.295 |
| CAD, n (%) |  |  | <0.001 |
| *1-vessel disease* | 52 (9.4) | 371 (26.3) |  |
| *2-vessel disease* | 111 (20.1) | 330 (23.4) |  |
| *3-vessel disease* | 390 (70.5) | 701 (49.8) |  |
| New- generation DES, n (%) | 533 (83.9) | 1203 (72.3) | <0.001 |

Data are shown as counts (%). Data were analyzed at a patient-level.

ACS= acute coronary syndrome; CAD= coronary artery diasease; MI= myocardial infarction

## **Supplemental Table 14. Ten-year clinical outcomes according to insulin therapy among patients with DM diagnosis at baseline.**

| Endpoint | Patients  Noninsulin treated  (n=1663) | Patients  Insulin treated  (n=635) | HR  (95% CI) | *P* value | HRadj  (95% CI) | *P* value |
| --- | --- | --- | --- | --- | --- | --- |
| All-cause death, n(%) | 573 (36.6) | 321 (53.6) | 1.75 (1.53-2.00) | <0.001 | 1.86 (1.69-2.04) | <0.001 |
| Cardiovascular death, n(%) | 322 (20.6) | 185 (31.0) | 1.81 (1.51-2.16) | <0.001 | 1.77 (1.52-2.07) | <0.001 |
| Non-cardiovascular death, n(%) | 251 (16.0) | 136 (22.6) | 1.67 (1.36-2.06) | <0.001 | 2.02 (1.82-2.24) | <0.001 |
| Myocardial infarction, n(%) | 138 (8.6) | 60 (9.8) | 1.26 (0.93-1.70) | 0.139 | 1.05 (0.93-1.18) | 0.415 |
| Definite ST, n(%) | 32 (2.0) | 12 (2.0) | 1.03 (0.53-2.01) | 0.923 | 0.94 (0.79-1.12) | 0.484 |
| Target lesion revascularization, n(%) | 291 (17.9) | 126 (20.4) | 1.29 (1.04-1.59) | 0.017 | 1.23 (1.13-1.34) | <0.001 |
| Target vessel revascularization, n(%) | 361 (22.1) | 169 (27.2) | 1.40 (1.16-1.68) | <0.001 | 1.32 (1.02-1.70) | 0.029 |
| Non-target vessel revascularization, n(%) | 401 (24.7) | 155 (25.1) | 1.16 (0.96-1.39) | 0.121 | 1.01 (0.82-1.25) | 0.886 |

HR: hazard ratio; ST: stent thrombosis; CI: confidence interval. Values are n (%). Kaplan-Meier estimates as percentages. Hazard ratios and 95% CIs are derived from cox proportional hazard models. Multivariable models were adjusted for age, body mass index, sex, smoke, DES generation, hypertension, hypercholesterolemia, prior myocardial infarction, acute coronary syndrome presentation, multivessel disease, and treatment of the left anterior descending coronary artery, with robust standard errors with clustering for trial.

# **SUPPLEMENTAL FIGURES**

## **Supplemental Figure 1. Ten-Year Cumulative Incidence of TLR (A), TVR (B), nTVR (C) according to DM diagnosis at baseline.**

## **Supplemental Figure 2. Landmark analysis for all-cause death (A), CV-death (B), MI (C), definite ST (D) according to DM diagnosis at baseline.**

## **Supplemental Figure 3. Landmark Analysis for myocardial infarction according to DM diagnosis at baseline.**

## **Supplemental Figure 4. Landmark Analysis for definite ST according to DM diagnosis at baseline**

## **Supplemental Figure 5. Trial-specific and pooled estimates of the association between DM diagnosis at baseline and all-cause death over 10 years.**

## **Supplemental Figure 6. Interaction between DM and cardiovascular death after 10 years among subgroups of patients treated with PCI and DES implantation.**

# **Supplemental Figure 7. Interaction between DM and myocardial infarction after 10 years among subgroups of patients treated with PCI and DES implantation.**

# **Supplemental Figure 8. Interaction between DM and stent thrombosis after 10 years among subgroups of patients treated with PCI and DES implantation.**

# **Supplemental Figure 9. Interaction between DM and target lesion revascularization after 10 years among subgroups of patients treated with PCI and DES implantation**

## **Supplemental Figure 10. Interaction between DM and target vessel revascularization after 10 years among subgroups of patients treated with PCI and DES implantation.**

## **Supplemental Figure 11. Interaction between DM and non- target vessel revascularization after 10 years among subgroups of patients treated with PCI and DES implantation.**
